# **40 years of** ***LEUKEMIA***

**DOI:** 10.1038/s41375-025-02840-y

**Published:** 2025-12-08

**Authors:** Andreas Hochhaus, Robert Peter Gale

**Affiliations:** 1 Klinik für Innere Medizin II, Universitätsklinikum Jena, Jena, Germany; 2 Centre for Haematology, Department of Immunology and Inflammation, Imperial College of Science, Technology and Medicine, London, UK

With this issue *LEUKEMIA* enters its 40th volume - a fitting moment to reflect on the journals history and impact.

*LEUKEMIA* was founded in 1987 by Nicole Muller-Bérat Killman and Sven-Aage Killman. It was initially published by Stockton Press under Macmillan Publishers and, since 2000, by the Nature Publishing Group (now Springer/Nature) with its editorial office in London. Nicole Muller-Bérat, who led the journal for 28 years, was the heart and soul of *LEUKEMIA* until her death in 2014 [1].

*LEUKEMIA* spans all aspects of leukemia research from basic science (molecular biology, genomics, proreomics, stem cells, oncogenes, signal transduction, normal and abnormal hematopoiesis) to clinical trials. Over 40 years it has evolved from focusing predominantly on basic sciencee in the 1990s to translational and clinical research in the 2000s including measurable residual disease (MRD), targeted therapies, and immune therapies. *LEUKEMIA* has become an important platform for consensus statements, clinical practice guidelines, methodological standards and high-impact clinical trials.

The first paper published in *LEUKEMIA* reported the responsiveness of myeloid cells to GM-CSF or G-CSF - a major topic in the 1980s [2]. Since then, 14 articles have received >1000 citations [3–16]. These landmark publications have shaped hematologic oncology bridging molecular mechanisms, diagnostics, and clinical practice. The current impact factor is 13.4.

WHO classification papers on myeloid, histiocytic/dendritic, and lymphoid neoplasms [3, 4] solidified *LEUKEMIA*’s role as a global authority in diagnostic standards. Foundational methodology contributions including BIOMED-2 PCR standardization [5] and Europe Against Cancer guidelines for RT-PCR–based MRD monitoring [6] position *LEUKEMIA* at the forefront of laboratory harmonization. Seminal clinical studies, such as International Myeloma Working Group response criteria [7] and analyses showing improved plasma cell myeloma survival [8], further reinforced the journal’s impact on clinical practice.

Key discoveries in leukemia biology, including *FLT3*-ITD mutations in AML [9], proposals for the immune classification of acute leukemias [10], genomic mapping of myelodysplastic neoplasia [11], and γH2AX as a DNA damage marker [12], cemented *LEUKEMIA*’s standing in translational research. European LeukemiaNet (ELN) recommendations [13, 17] shaped global standards for chronic myeloid leukemia management while pioneering work on microvesicle-mediated intercellular communication [14, 15] broadened its impact across stem cell, cancer, and immunology fields. Collectively, these contributions underscore *LEUKEMIA*’s central role in advancing classification, biology, diagnosis, and therapy in hematological cancers.

We were honored to become Editors-in-Chief in 2014. Alongside our international team of 26 Associate Editors and 45 Editorial Board members we look forward to a successful 40th volume and encourage submissions of Original Articles, Reviews, Letters, Perspectives, and Commentaries. Correspondence to published articles is always welcome. We encourage debate. Ms. Lucinda Haines has been our Publishing Editor with Springer/Nature since 2009. *LEUKEMIA* continues to welcome work that advances basic and clinical research in leukemia and related disorders.
